# Legal Notes

**Published:** 1899-12-23

**Authors:** 


### LEGAL NOTES.

### COMMISSIONERS IN LUNACY versus PHILIP HENRY HARMER.

This case, in which the defendant was charged with receiving a lunatic, and also with unlawfully receiving payments for a lunatic in an unlicensed house, was tried before the magistrates at Mark's Cross, Sussex, on November 14th. The patient, a medical man, was sent to South Beacon, Hadlow Down, on May 2nd, having locomotor ataxia. The case seems to have been one of those which at last drift into insanity, and it must always be extremely difficult in these cases to say positively that the line between sanity and insanity has been crossed. Probably in this case the difficulty was greater than usual, as the patient was one of those men who study and brood over their symptoms. Dr. Gaveney and Dr. Hall attended, and gave evidence that he was sane up to about the middle of August. He was certified insane on the 27th, and was taken to an asylum on September 2nd. There are here about twelve days during which Mr. Harmer kept the patient after he knew he was insane—surely not a very long time for his friends to make arrangements for his removal. There was no charge of ill-usage. On the contrary, the patient's wife said she was fully satisfied with the treatment her husband had received at South Beacon, and it may be pointed out that had the strict letter of the law been carried out on August 15th (the first certifiable day according to Dr. Hall) the poor patient would have been handed over to the relieving officer, a most cruel position to place a gentleman in. The case occupied six hours in hearing, and the Bench dismissed it. It is not easy to see what other decision they could have arrived at, but it would not be unreasonable to inquire what compensation Mr. Harmer will get for the expense and trouble he has been put to? We might further ask if the Commissioners in Lunacy have power to institute these proceedings without reference to any other authority, and has the country to pay the cost of these unsuccessful prosecutions? The Commissioners in Lunacy for Scotland say they "do not regard it as desirable that any class of persons should be brought under official supervision, unless such supervision appears to be necessary to guard against abuse." The consequence of this enlightened view is that such prosecutions as are common in England are rarely heard of in Scotland; and it is beyond doubt that the Scotch lunatic has greater personal freedom than his English brother. There are very numerous cases on the borderland of insanity who are not certifiable strictly speaking, and whose friends would refuse to think of such a proceeding, who would nevertheless immensely benefit by treatment in a private house away from their relatives. But what medical man would take these cases when he may have to defend an action for breach of the Lunacy Act. As usual the unfortunate patient suffers, and actual insanity sooner or later declares itself.

